# Blue and Far-Red Light Affect Area and Number of Individual Leaves to Influence Vegetative Growth and Pigment Synthesis in Lettuce

**DOI:** 10.3389/fpls.2021.667407

**Published:** 2021-07-08

**Authors:** Yuyao Kong, Krishna Nemali

**Affiliations:** Department of Horticulture and Landscape Architecture, Purdue University, West Lafayette, IN, United States

**Keywords:** controlled environment agriculture, photoinhibition, phytochrome photostationary state, light interception, proximity response

## Abstract

Published work indicates that high percentage of blue light can enhance pigment levels but decreases growth, while addition of far-red light to growth light can increase quantum efficiency and photosynthesis in leafy greens. Combining high-energy blue light with low-energy far-red light may increase both vegetative growth and pigment levels. However, the effect of high-energy blue and low-energy far-red light on the vegetative growth and pigments synthesis is unclear. This information can be potentially useful for enhancing the levels of pigments with nutritional value (e.g., beta-carotene and anthocyanins) in the produce grown in vertical farms. We grew romaine lettuce (cv. Amadeus) under similar light intensity (approximately 130 μmol⋅m^–2^⋅s^–1^) but different proportions of red: blue: far-red including 90:10: 0 (“High-R”), 50: 50: 0 (“High-B”), and 42: 42: 16 (“High-B+FR”) for 31 days. Results indicated that canopy area and leaf photosynthetic rate of lettuce plants was reduced in the High-B, thereby reducing plant growth. We did not observe photosynthesis enhancement in the High-B+FR. Instead, plants clearly showed photomorphogenic effects. The phytochrome photostationary state (PSS) decreased with far-red addition, resulting in reduced leaf number per plant. This was likely to shift the allocation of resources toward elongation growth for shade avoidance. Further, we observed an increase in the area of individual leaves, canopy area, and shoot dry weight in the High-B+FR. However, these appear to be an indirect consequence of decreased leaf number per plant. Our results also indicate that changes in expansion growth at individual leaf scale largely regulated pigment concentration in plants. As individual leaf area became smaller (e.g., High-B) or larger (e.g., High-B+FR), the levels of pigments including chlorophylls and beta-carotene increased or decreased, respectively. Area of individual leaves also positively influenced canopy area (and likely light interception) and shoots dry weight (or vegetative growth). Our study provides additional insights into the effects of high-energy blue and low-energy far-red light on individual leaf number and leaf growth, which appear to control plant growth and pigment levels in lettuce.

## Introduction

Vertical farming involves growing food crops at multiple vertically stacked levels in controlled environments to maximize productivity per unit area (Despommier, 2010). The concept of vertical farming was mainly developed to produce food in urban areas where available space can be limited for growing crops. Vertical farming can aid in increasing the supply of fresh food, which is needed in many urban areas (Avgoustaki and Xydis, 2020). Because of its importance to food production, the industry is growing rapidly in the United States, with an estimated market value of $3 billion by 2024 (Qiu et al., 2020). At present, leafy greens such as lettuce are the major crops grown in vertical farms (Frazier, 2017). With developments in science and technology, the range of species, including vegetables, medicinal plants, and ornamentals, that can be grown in vertical farms is expected to increase in the future.

Plant pigments such as beta-carotene (precursor of vitamin A biosynthesis) and anthocyanins (aid in reducing inflammation and blood pressure, Karlsen et al., 2007; Jennings et al., 2012) have beneficial effects on human health. Nutrients consumed through plant-based foods are regarded as more efficacious than those consumed through supplements (Martin and Li, 2017). However, the levels of nutrients beneficial to human health are generally low in plants (Lako et al., 2007; Thoma et al., 2020). For example, a salad bowl of lettuce (approximately 60 g) can provide only a third of recommended daily allowance of vitamin A (Institute of Medicine US Panel on Micronutrients, 2001; USDA, 2019). Breeding efforts to improve the levels of nutrients in leafy greens is at its infancy. The current research focus in the area of vertical farming is on increasing crop productivity and optimizing resource-use during production. Relatively less effort is being made to explore the potential of vertical farms to supply nutrient-dense foods to urban population. Given this, environmental manipulation appears to be the only viable option for enhancing the level of healthy nutrients in leafy greens.

Light emitting diode (LED) lights with a wide range of customized light spectra are used to grow plants in vertical farms (Kong et al., 2019). Spectral composition of light can significantly affect pigment levels in plants (Amoozgar et al., 2017; Naznin et al., 2019; Camejo et al., 2020). Addition of high-energy blue light is usually associated with increased accumulation of pigments such as carotenoids and anthocyanins (Li and Kubota, 2009; Johkan et al., 2010; Ouzounis et al., 2015; Amoozgar et al., 2017), and chlorophylls (Cope et al., 2013; Fan et al., 2013; Zhen and Bugbee, 2020). However, blue light is less efficient at driving photosynthesis than red light (McCree, 1972). Plants exposed to excess blue light show decreased leaf expansion (Hernandez and Kubota, 2016; Craver et al., 2020; Kusuma et al., 2020), stem elongation, and growth (Ohashi-Kaneko et al., 2007; Fan et al., 2013; Kang et al., 2016; Craver et al., 2020). Blue light is also involved, *via* cryptochrome receptors, in de-etiolation of hypocotyls and photoperiodic responses related to flowering (Yu et al., 2010), and phototropism (Ahmad et al., 1998) in plants. Far-red light addition enhanced quantum efficiency (or efficiency with which absorbed light is used in photosynthesis) when supplemented with light containing shorter wavelengths of photosynthetically active radiation (Zhen and van Iersel, 2017). It was reported that the absorbed far-red photons (700–750 nm) were equally efficient for photosynthesis when combined with photosynthetically active radiation (400–700 nm; Zhen and Bugbee, 2020). Increases in leaf area and canopy light interception (Park and Runkle, 2017; Kang et al., 2019; Zhen and Bugbee, 2020) were reported with far-red addition. However, far-red addition resulted in a decrease in the levels of carotenoids and anthocyanins (Li and Kubota, 2009; Stutte and Edney, 2009), and chlorophylls (Meng and Runkle, 2019; Zhen and Bugbee, 2020).

Moreover, far-red light has been associated with photomorphogenic effects associated with shade avoidance (e.g., stem elongation) in plants (Ballaré et al., 1990; Gommers et al., 2012; Yang and Li, 2017). A small increase in the proportion of far-red light in the environment can elicit “proximity” shade responses in plants, prior to actual canopy shading by neighbors (Martinez-Garcia et al., 2014). Based on the published research (Lorrain et al., 2008; Martinez-Garcia et al., 2014; Yang and Li, 2017), shade stimulus is perceived by phytochrome (P) receptor. The receptor exists in two photo-convertible forms i.e., red light absorbing P_r_ (inactive) and far-red light absorbing P_fr_ (active) form. The P_r_ form converts to P_fr_ upon absorption of red light and the P_fr_ form reverts to P_r_ form after absorbing far-red light or in the dark. Among the important light signaling components, phytochrome interacting factors (PIFs) play an important role in shade avoidance response. The activation of PIFs is necessary for eliciting shade avoidance responses such as elongation. A lower proportion of P_fr_ form in plants results in activation of PIFs and subsequent gene expression required for shade avoidance responses. This happens when relatively more P_fr_ converts to P_r_ form, i.e., when plants are exposed to far-red light.

Addition of low-energy far-red light to growth spectrum containing relatively high proportion of high-energy blue light may increase photosynthesis, crop growth rate, and pigment concentration in the produce grown in vertical farms. However, addition of far-red light may elicit photomorphogenic responses including elongation and may have little direct impact on photosynthesis in the long-term. Shade-avoidance responses due to far-red exposure may lower levels of pigments in the cells, as additional resources are needed for the elongation growth. Further, elongation growth may decrease plant quality. The interactive effects of high-energy blue light and low-energy far-red light on vegetative growth and pigment levels in plants is unclear. This information is important to exploit the benefits of blue and far-red spectrum in vertical farming. The objectives of the study were to quantify the effects of blue and combination of blue and far-red spectrum on vegetative growth and pigment concentration and to understand how light composition affects the interplay between vegetative growth and pigment synthesis in lettuce plants.

## Materials and Methods

### Plant Production, Growing System, and Environmental Conditions

Romaine lettuce (*Lactuca sativa* cv. Amadeus) was grown from seed purchased from Paramount Seeds Company (Stuart, FL, United States). The selected variety has green foliage (i.e., contain low level of anthocyanins) and low crop yield (Miller et al., 2020), making it suitable for studying treatment effects on pigment levels and vegetative growth. Seeds were germinated in plug flats (72-cell; 3.5 cm × 3.5 cm × 5.9 cm, 30.2 mL per cell, Landmark Plastic, Akron, OH, United States) filled with a soilless substrate (80% peat, 15% perlite, and 5% vermiculite, BM-2, Berger, Saint Modeste, QC, Canada). The plug flats were placed under mist for 7 days to ensure uniform germination. Light treatments started immediately after germination (see section “Treatments”). After 10 days, seedlings in each light group were transplanted into square pots (10.6 cm × 10.6 cm × 8.4 cm, 943 mL, Kord Products Ltd., Brampton, ON, Canada) filled with the same soilless substrate used in the germination trays and arranged in a predetermined experimental design (see section “Experimental Design and Statistical Analyses”). Plants were harvested after 31 days from transplanting.

**FIGURE 1:**
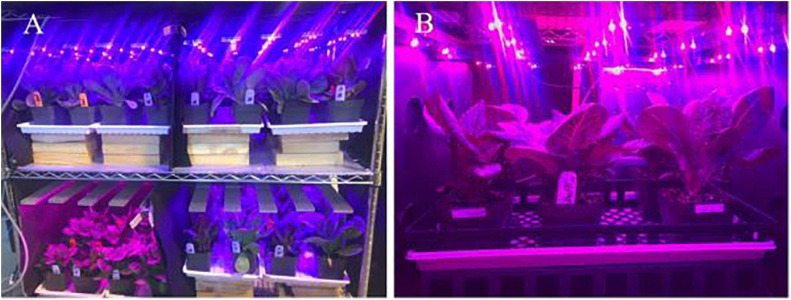
**(A)** Experimental setup used in the study showing different experimental units. **(B)** A closeup picture of one experimental unit.

The vertical growing system was custom-built using chrome-wire shelves (1.22 m × 0.61 m × 1.37 m, H-6948, Uline, Pleasant Prairie, WI, United States) and LED fixtures. Each shelf had two levels spaced 0.6 m apart. Each level was further divided into two grow spaces (0.61 m × 0.61 m, of 0.37 m^2^) and each grow space housed plants in pots (Figure 1). Customized LED fixtures (0.6 m × 0.6 m, Applied Electronic Materials, Fort Wayne, IN, United States) with different spectral composition were fastened to the chrome-wire shelves in each grow space. The light fixtures had separate circuits for blue (450 ± 18 nm), red (660 ± 19 nm), and far-red (730 ± 30 nm) LEDs (Oslon SSL, Osram, Munich, Germany). Each light fixture comprised of five individual bars (60 cm long), each containing six LEDs of a given wavelength. The LEDs were spaced 10 cm apart in the circuit and emitted light at 120° angle from the source. The intensities of red, blue, and far-red wavebands emitted from each fixture were adjusted using a controller (Time-Keeper MAX, Touch-Plate Light Controls, Fort Wayne, IN, United States). The LED fixtures were hung 0.5 m above the pots in each grow space.

Plants were grown using sole-source lighting from the LED fixtures. As the vertical growing system was located in a greenhouse, it was covered with two layers of black cloth (WeedBlock, Jobes Co., Waco, TX, United States) that allowed air movement but blocked sunlight to ensure a sole-source LED lighting environment for plants. Average day/night air temperature, daily light integral, photoperiod, and relative humidity during the study were 21.7 ± 1.43/19.5 ± 0.81°C, 11.4 ± 0.17 mol⋅m^–2^⋅d^–1^, 24 h, and 62 ± 12.5%, respectively. Overhead irrigation with a fertilizer solution was provided as needed during the production to avoid any drought stress. Plants were fertigated with a water-soluble fertilizer containing 20N-4.4P-16.6K (20-10-20, Peters Professional, Summerville, SC, United States) at an electrical conductivity (EC) level of 1.7 ± 0.04 dS⋅m^–1^ and pH of 5.8 ± 0.04.

### Treatments

**FIGURE 2:**
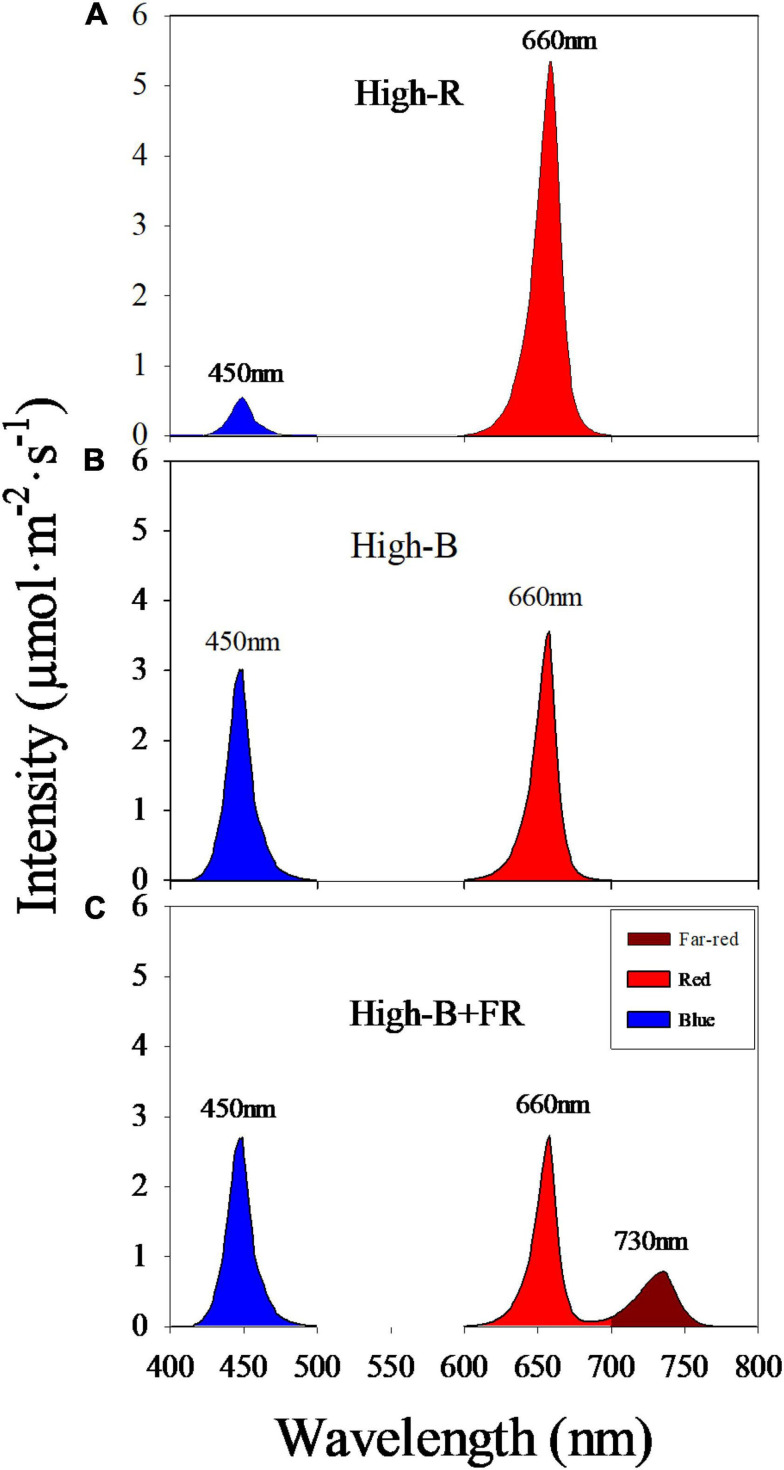
Spectral composition in different light treatments: **(A)** red: blue: far-red = 90: 10: 0 (“High-R”), **(B)** red: blue: far-red = 50: 50: 0 (“High-B”), and **(C)** red: blue: far-red = 42: 42: 16 (“High-B+FR”). Peak wavelength numbers are shown for each broadband.

Plants were grown under three light treatments with different spectral composition and similar light intensity (Figure 2 and Table 1). Light treatments comprised of different percentages red: blue: far-red light in the total light including 90: 10: 0 (“High-R”), 50: 50: 0 (“High-B”), and 42: 42: 16 (“High-B+FR”). Black plastic sheets (0.61 m × 0.4 m, HomeDepot, Atlanta, GA, United States) were used to separate the grow spaces in each shelf and contain the dispersed light within each treatment. The total light intensity (400–750 nm) ranged between 129 and 138 μmol⋅m^–2^⋅s^–1^ in different treatments (Table 1).

**TABLE 1:** Total light intensity (400–750 nm), phytochrome photostationary state (PSS), and spectral composition of light in different treatments.

**Treatment**	**Intensity**	**PSS**	**Spectral composition**		
			**Blue**	**Red**	**Far-red**
	**(μmol⋅m^–2^⋅s^–1^)**		**(%)**		
High-R	128.9 (1.67) a	0.90 (0.0008) a	8.3 (0.39) c	91.7 (0.39) a	0 (0.00) b
High-B	132.2 (1.44) a	0.86 (0.0008) b	47.2 (0.24) a	52.8 (0.24) b	0 (0.00) b
High-B+FR	138.1 (1.52) a	0.78 (0.0008) c	41.6 (0.20) b	41.5 (0.28) c	16.9 (0.12) a

*Percentages of photons associated with broadband blue (400–500 nm), red (600–699 nm), and far-red (700–750 nm) light are shown. Values represent least square means and standard errors (±) (in parenthesis).*

### Measurements

Air temperature (°C) was measured in each experimental unit (see section “Experimental Design and Data Analyses”) using thermistors (ST-110, Apogee Instruments, Logan, UT, United States) connected to a data logger (CR1000, Campbell Sci. Logan, UT, United States). Total incident light intensity (I_tot_, μmol⋅m^–2^⋅s^–1^) and spectral composition were measured at plant height using a spectroradiometer (SS-110, Apogee Instruments, Logan, UT, United States) with a hemispherical field-of-view (180°). From spectroradiometer measurements, broadband intensities of blue, red, and far-red wavelengths (I, μmol⋅m^–2^⋅s^–1^) were calculated by adding intensities of individual wavelengths between 400–499 nm, 600–699 nm, and 700–750 nm, respectively. Percentage of blue, red, and far-red photons in the total light was calculated as $100\times \frac{\text{I}}{{\text{I}}_{\text{tot}}}.$

The proportion of P_fr_ to total phytochrome in plants was calculated as phytochrome photostationary state (PSS) using spectral composition and intensity of light received by plants (Sager et al., 1988; Stutte, 2009):

$$\mathrm{PSS}=\frac{\sum_{400}^{750}\left(N_{\lambda }\sigma_{r\lambda }\right)}{\left(\sum_{400}^{750}{\text{N}}_{\lambda }\sigma_{r\lambda }+\sum_{400}^{750}{\text{N}}_{\lambda }\sigma_{\mathrm{fr}\lambda }\right)}$$

Where *N*_*λ*_ is light intensity (mol⋅s^–1^⋅m^–2^) at each wavelength (λ). The σ_*r*_λ__ and σ_*fr*_λ__ are phytochrome photochemical cross-sections for red and far-red absorbing states (m^2^⋅mol^–1^) at each λ based on measurements made by Sager et al. (1988) on isolated phytochrome. The λ levels used in the calculation were 400, 425, 450, 475, 500, 600, 625, 660, 675, 700, 725, 730, and 750 nm. The *N*_*λ*_ was measured by a spectroradiometer (SS-110, Apogee Instruments, Logan, UT, United States).

Leaf photosynthetic rate (LPR, μmol⋅m^–2^⋅s^–1^) was measured as described by Long et al. (1996) on the youngest fully expanded leaf during the last week before harvest using an open gas exchange system with CO_2_, humidity, temperature, and light control (LI-6400XT, Li-Cor Biosciences, Lincoln, NE, United States). A clear-top leaf chamber was used to measure leaf gas exchange rate in different light treatments. This allowed us to measure leaf gas exchange under similar spectral composition and intensity of light incident on the plants in a given treatment. A reference CO_2_ concentration of 400 μmol⋅mol^–1^, RH of 60%, and air temperature of 25°C were maintained during the measurement inside the leaf chamber. Measurements were recorded when gas exchange reached steady state, which occurred between 2 and 3 min after enclosing the leaf inside the chamber.

A representative plant from the center of each experimental unit was used to measure canopy area (CA, cm^2^⋅plant^–1^), leaf area (LA, m^2^⋅plant^–1^), shoot fresh weight (SFW, g⋅plant^–1^), and shoot dry weight (SDW, g⋅plant^–1^) at harvest. CA was measured as described by Adhikari and Nemali (2020) on the 31st day of the study using an imaging system (TopView phenotyping system, Aris B.V. Eindhoven, Netherlands). The distance between the camera and top of the plant was maintained similar during measurements. The software of the image station automatically segmented plants from the background, measured plant pixel area, and converted plant pixel area to CA by multiplying with a magnification factor (100) specific to the imaging system. Number of leaves on each plant (L_N_) was counted prior to harvest. Plants were harvested at the base of the shoot and SFW was measured. Leaves were separated from plants and LA was measured by running separated leaves through the rollers of a leaf area meter (LI-3100C, Li-Cor Biosciences, Lincoln, NE, United States). Area of a single leaf (LA_s_, m^2^) was calculated as the ratio of total leaf area to leaf number. Separated leaves and remaining plant material were dried in a forced-air oven set to 70°C for 1 week to measure SDW.

Levels of chlorophylls (Chl), including chlorophyll-*a* (Chl_*a*_) and chlorophyll-*b* (Chl_*b*_), and beta-carotene (β-carotene) were measured as described by Nagata and Yamashita (1992). Plant samples collected from each experimental unit was used to analyze plant pigments at harvest stage. Two mature leaves from a plant were flash-frozen in liquid nitrogen and ground into fine power using a mortar and pestle. Approximately 0.2 g of the ground tissue was extracted with 1.8 ml acetone-hexene (2:3, v/v) solvent until the tissue turned white. Then the samples were centrifuged at 12,000 rpm for 3 min using a benchtop centrifuge (Sorvall Legend Micro 21 micro-centrifuge, Thermo Fisher Scientific, Waltham, MA, United States). The supernatant was then applied to a 1.4 ml quartz cuvette (Fisher Scientific, Waltham, MA, United States) to measure the absorption at 663, 645, 505, and 453 nm using a spectrophotometer (GENESYS 180 UV-Vis, Thermo Fisher Scientific, Waltham, MA, United States). The concentration of pigments was calculated on a fresh weight basis (mg⋅100 g^–1^) as follows:

Chl_*a*_ = [0.999 × A_663_] − [0.0989 × A_645_]
Chl_*b*_ = [−0.328 × A_663_] + [1.77 × A_645_]
Chl = Chl_*a*_ + Chl_*b*_
β-carotene = [0.216 × A_663_] − [1.22 × A_645_] −
[0.304 × A_505_] + [0.452 × A_453_]

Anthocyanins were extracted from the same leaf samples used for Chl and β-carotene analyses. A total of 0.1 g of the ground tissue was extracted using 4 ml of pre-cooled (4°C) 1% HCL-methanol solution (v/v) in tubes covered with aluminum foil, and placed in a refrigerator (dark) maintained at 4°C for 20 min. The mixed solution in the tubes was shaken several times during extraction. Later it was centrifuged at 12,000 rpm for 3 min using a benchtop centrifuge (Sorvall Legend Micro 21 microcentrifuge). The supernatant was then applied to 1.5 ml cuvettes (Fisher Scientific, Waltham, MA, United States). Optical density (OD) were measured at 530 nm (OD_530_) and 600 nm (OD_600_) using the spectrophotometer (GENESYS 180 UV-Vis). The difference of OD between 530 and 600 nm was used to estimate a relative measure of anthocyanins concentration on a fresh weight basis (ΔOD⋅100 g^–1^) as follows:

Anthocyanins = (OD_530_-OD_600_)

Concentration of above pigments was also expressed on a leaf area basis (mg or ΔOD × m^–2^) as follows:

$$\left[{\text{Pigment}}_{\mathrm{area}\mathrm{basis}}\right]=\frac{\left[{\text{Pigment}}_{\mathrm{fresh}\mathrm{weight}\mathrm{basis}}\right]}{100}\times \frac{\text{FW}}{\text{LA}}$$

### Experimental Design and Statistical Analyses

There were two experiments in the study. Both experiments were laid out using a randomized block design. Light treatments were randomly allotted in each replication. An experimental unit comprised of a set of three plants belonging to a light treatment, replicated three and four times in experiments 1 and 2, respectively. Photosynthesis measurements were made in experiment 1 while Anthocyanin and L_N_ were measured in experiment 2. All the other measurements were made in both the experiments. Data were analyzed using a linear-mixed model (MIXED procedure) of statistical analysis software (SAS ver 9.4, Cary, NC, United States). Treatments were considered as fixed effects while both replications and experiments were considered as random effects in the model. Least-square means were separated using Tukey’s honestly significant difference (HSD) procedure. Relationship between any two variables was tested using both the linear and quadratic regression procedures of SAS. A pre-determined alpha value of 5% (*P*-value ≤ 0.05) was considered statistically significant for all analyses.

## Results and Discussion

### Light Spectral Composition

Average instantaneous light intensity received by the plants was not statistically different and within 10 μmol⋅m^–2^⋅s^–1^ among different light treatments (Table 1). However, spectral composition significantly varied among different light treatments (Figure 2). The actual percentages of red, blue, and far-red light were slightly different from the intended treatment values (Table 1). Further, small standard errors of least square means indicate that total light intensity and light composition were highly consistent among different replications belonging to a light treatment. The PSS values were significantly different among the three light treatments, with highest in High-R, followed by High-B, and lowest in the High-B+FR. This indicates that phytochrome equilibrium differed among treatments and suggests that the relative amount of P_fr_ to total phytochrome was likely highest in the High-R, followed by High-B, and lowest in the High-B+FR treatment.

### Morphological, Growth, and Physiological Differences

**FIGURE 3:**
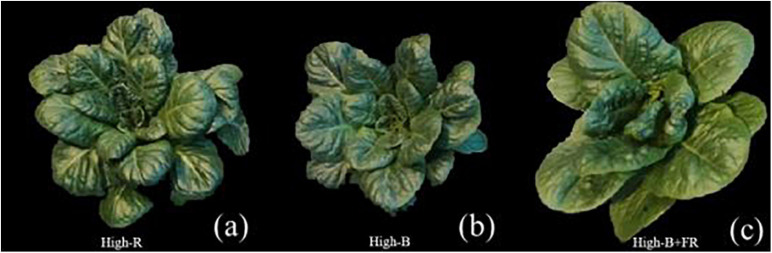
Representative romaine lettuce plants from different light treatments. **(a)** High-R, **(b)** High-B, and **(c)** High-B+FR treatments. See Table 1 for a description of treatments.

Plants appeared visually different in the High-B and High-B+FR treatments compared to High-R treatment (Figure 3). Plants and individual leaves were generally smaller in the High-B treatment compared to High-R and High-B+FR treatments. These differences may indicate lower LPR and/or light interception in High-B treatment. In the High-B+FR treatment, plants had both elongated and expanded leaves. In addition, plants were pale with fewer leaves in the High-B+FR compared to the other treatments. The canopy was open with relatively less intra-canopy shading in the High-B+FR treatment. These phenotypic characteristics may indicate that plants in the High-B+FR exhibited shade avoidance responses.

Shoot fresh weight was significantly higher in the High-R than High-B and High-B+FR treatments (Table 2). There were no significant differences in SFW between High-B and High-B+FR treatments. The SFW of plants increased by 9.8 and 8.8%, respectively, in the High-R compared to High-B and High-B+FR. Whereas SDW of plants was significantly lower in the High-B treatment compared to the other treatments and not different between High-R and High-B+FR treatments. The increase in SDW of plants in the High-B+FR and High-R compared to High-B was 13% and 18%, respectively. A decrease in both SFW and SDW of plants in the High-B compared to High-R indicates slower growth rate in the High-B treatment. Further, the results indicate that reduced growth rate in High-B was reversed by the addition of far-red light. No differences in SFW but an increase in SDW in the High-B+FR than High-B suggest increased shoot water content in High-B than High-B+FR. Similarly, decrease in SFW in High-B+FR but no differences in SDW between High-B+FR and High-R suggest a decrease in shoot water content of plants in the High-B+FR compared to High-R.

**TABLE 2:** Effect of different light treatments (see Table 1 for treatment description) on shoot fresh weight (SFW), shoot dry weight (SDW), leaf area (LA), canopy area (CA), leaf photosynthesis rate (LPR), leaf number (L_N_), and average area of *a* single leaf (LA_s_) in green romaine lettuce.

**Measurement**	**Treatment**		
	**High-R**	**High-B**	**High-B+FR**
SFW (g⋅plant^–1^)	151.7 (7.09) a	136.9 (7.09) b	138.2 (7.09) b
SDW (g⋅plant^–1^)	9.8 (0.52) a	8.1 (0.52) b	9.0 (0.52) a
LA (m^2^⋅plant^–1^)	0.208 (0.0100) a	0.191 (0.0100) a	0.182 (0.0100) b
log_10_(CA)	1.96 (0.056) a	1.81 (0.056) b	1.94 (0.056) a
LPR (μmol⋅m^–2^⋅s^–1^)	4.5 (0.25) a	3.6 (0.25) a^1^	3.5 (0.25) a^2^
L_N_ (plant^–1^)	19.7 (0.88) a	18.7 (0.88) a	11.3 (0.88) b
LA_s_(m^2^)	0.008 (0.0009) b	0.007 (0.0009) b	0.012 (0.0009) a

*Least square means and standard errors (±) (in parenthesis) are shown. Least square means followed by different letters are statistically different at *P* ≤ 0.05.^1^*P*_High–R vs High–B_ = 0.062.^2^*P*_High–R vs High–B + FR_ = 0.052.*

Leaf area of plants was not different between the High-R and High-B and significantly lower in the High-B+FR treatment (Table 2). These results indicate a lack of association between LA and SDW. Although LPR was not statistically different among treatments, it was numerically higher in the High-R compared to High-B (*P* = 0.062) and High-B+FR (*P* = 0.052) (Table 2). There were no statistical differences in LPR between High-B and High-B+FR treatments. Further, this indicates that the addition of far-red light to high percentage of blue light did not “enhance” LPR of lettuce in the long-term. In addition, the results indicate that LPR is highly sensitive to high-energy blue light as lower LPR was observed in both treatments with high percentage of blue light (i.e., High-B and High-B+FR). However, CA responses were similar to those observed for SDW. Log transformed CA was not significantly different between the High-R and High-B+FR treatments but significantly lower in the High-B treatment, similar to that of SDW. A lower CA in the High-B treatment may suggest that plants intercepted less incident light and an increase in CA in the High-B+FR compared to High-B indicate that plants intercepted more incident light.

Vegetative growth is related to both light interception by the canopy and light use in photosynthesis (He et al., 2019; Liao et al., 2019). Because LA, CA, and LPR were highest, maximum vegetative growth was observed in the High-R treatment (Table 2). Other studies also reported that high percentage of red light can efficiently drive photosynthesis, promote leaf expansion, stem elongation, and dry mass gain in plants (Gómez and Izzo, 2018; Kusuma et al., 2020). Shoot growth was lowest in the High-B treatment likely due to significant decrease in CA and numerical decrease in LPR, compared to the High-R treatment. The CA measurement in our study is proportional to the non-shaded leaf area. In other studies, CA was related to light interception (Zhen and Bugbee, 2020) and biomass production (Zhu et al., 2010; Jones et al., 2015) in plants. Thus, lower CA likely contributed in part to decreased growth in the High-B than High-R. Blue light was found to be photosynthetically less efficient than red light (McCree, 1972; Cope et al., 2013; Kusuma et al., 2020). Therefore, lower LPR also likely contributed to decreased growth in the High-B than High-R. Although LPR decreased in High-B+FR, an increase in CA was observed in this treatment suggesting increased light interception (Table 2). The absorbed far-red photons (700–750 nm) can be equally efficient for photosynthesis when combined with photosynthetically active radiation (400–700 nm) (Zhen and Bugbee, 2020). Collectively these can explain increased dry weight in the High-B+FR compared to High-B.

The percentage of blue light in the High-B (47.2%) was much higher than the recommended level of 10–15% of total light (Hoenecke et al., 1992; Son and Oh, 2013; Ouzounis et al., 2014; Runkle, 2016). High-energy blue light was reported to increase oxidative stress (Ohnishi et al., 2005), resulting in damage to photosynthetic machinery. Anthocyanins are synthesized in the cytosol (Tanaka et al., 2008), absorb blue and green wave bands (Pietrini and Massaeei, 1998; Kusuma et al., 2020), and screen photosynthetic machinery from damage due to high-energy radiation (Close and Beadle, 2003; Hughes et al., 2012). Anthocyanins function by attenuating the light that reaches chloroplasts (Gould et al., 1995; Mendez et al., 1999; Smillie and Hetherington, 1999; Close, 2001; Zhang et al., 2010). We observed no differences in anthocyanins between High-B and High-R and decrease between High-R and High-B+FR. It is possible that the decreases in LPR in High-B and High-B+FR treatments are likely due to a large fraction of photosynthetically less efficient blue radiation (McCree, 1972) in these treatments. Enhancement in LPR was reported with the addition of far-red light to broadband white light due to simultaneous activation of both photosystem II and I reaction centers (Zhen and van Iersel, 2017). There were no differences in LPR between High-B+FR and High-B, indicating no enhancement of photosynthesis due to addition of far-red light to high-energy blue radiation in our study. Chl_*a*_, an integral component of the reaction center of photosystem (Horton et al., 2002; Fromme et al., 2006), was significantly lower [based on Chl_(*a/b*)_ ratio, Table 3] in the High-B+FR than High-B. In addition, β-carotene levels were significantly lower in in the High-B+FR than High-B (Table 3). β-carotene is mostly present in the reaction center of photosystem (Härtel and Grimm, 1998; Ruiz-Sola and Rodríguez-Concepción, 2012) and aid in absorbing light in the range of 450–570 nm where Chl absorption declines (Ruiz-Sola and Rodríguez-Concepción, 2012). Collectively, these may have partly contributed to lack of photosynthesis enhancement in the High-B+FR treatment.

**TABLE 3:** Effect of different light treatments (see Table 1 for treatment description) on the levels of anthocyanins, beta-carotene (β-carotene), chlorophylls (Chl) and ratio of chlorophyll-*a* to chlorophyll-*b* [Chl_(*a/b*)_] in green romaine lettuce.

**Measurement**	**Treatment**					
	**High-R**	**High-B**	**High-B+FR**	**High-R**	**High-B**	**High-B+FR**
	**Fresh weight basis (ΔOD or mg⋅100 g^–1^)**			**Leaf area basis (ΔOD or mg⋅m^–2^)**		
Anthocyanins	0.012 (0.0037) a	0.013 (0.0037) a	0.009 (0.0037) a	0.069 (0.0181) a	0.072 (0.0181) a	0.051 (0.0181) a
β-carotene	5.98 (0.693) ab	6.99 (0.693) a	4.85 (0.693) b	46.1 (4.71) ab	50.7 (4.71) a	40.1 (4.71) b
Chl	63.6 (6.78) ab	70.7 (6.78) a	54.4 (6.78) b	453.1 (46.13) a	488.6 (46.13) a	406.8 (46.13) a^1^
Chl_(*a/b*)_	2.44 (0.063) a	2.42 (0.063) a	2.26 (0.063) b	–	–	–

*Data was expressed on both fresh weight and leaf area basis. Least square means and standard errors (±) (in parenthesis) are shown. Least square means followed by different letters are statistically different at *P* ≤ 0.05.*^1^*P*_High–B vs⋅ High–B+FR_ = 0.077.

### Leaf Number and Individual Leaf Area

**FIGURE 4:**
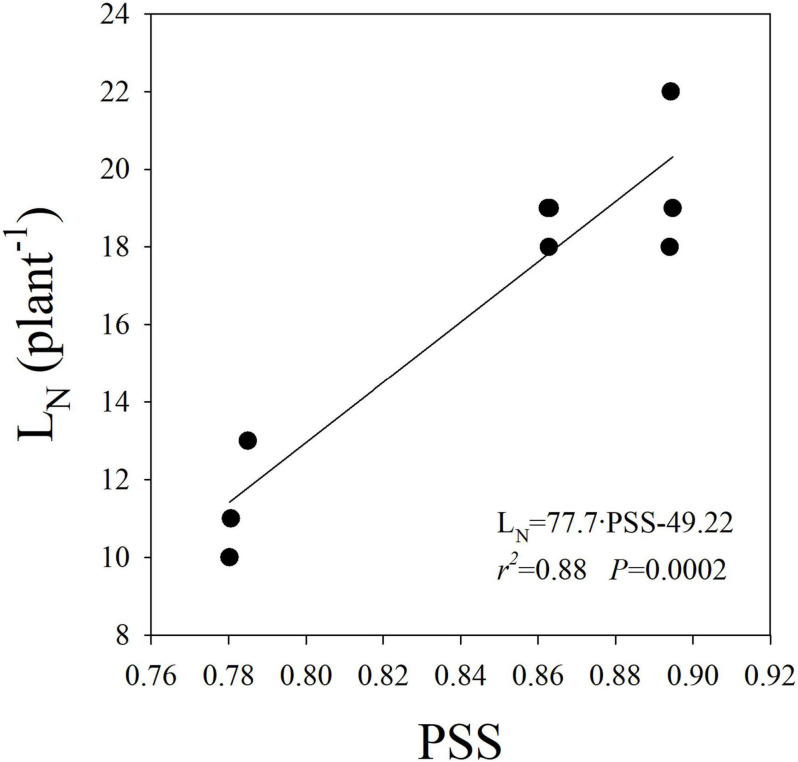
Linear relationship between leaf number (L_N_) and phytochrome photostationary state (PSS) in romaine lettuce. A linear regression was fitted to data from three light treatments and three replications per treatment.

A significant reduction in L_N_ was observed in the High-B+FR compared to High-B and High-R treatments (Table 2). The result indicates a decrease in leaf primordia in plants with the addition of far-red light. There was a linear and positive relationship between L_N_ and PSS (Figure 4), indicating that L_N_ increased with increasing P_fr_ to total phytochrome in plants. As P_fr_ increases with increasing red light incident on plants, the results indicate that high proportion of red light increases L_N_. Previously, decrease in L_N_ due to far-red addition in lettuce was reported (Meng and Runkle, 2019). There was no association between LA or CA and L_N_ (data not shown), indicating leaf development happened independent of leaf primordia number or initiation in plants. The linear and positive relationship between L_N_ and PSS (Figure 4) indicates that lower L_N_ in the High-B+FR is likely associated with lower phytochrome equilibrium or increased proportion of P_r_ to total phytochrome in the High-B+FR treatment due to increased far-red light. Plants use increased proportion of far-red light in the environment as an indicator of proximity of neighbors and respond by exhibiting suit of traits (e.g., elongation) that reduce future canopy shading (Ballaré et al., 1990; Gommers et al., 2012; Yang and Li, 2017). The “proximity response” can happen even due to a slight decrease in red to far-red ratio and does not require a reduction in the intensity of total or red light (Martinez-Garcia et al., 2014). Increased proportion of far-red light (which lowers PSS value) resulted in reduced leaf primordia (Carabelli et al., 2007; Yang and Li, 2017) and increased elongation growth (Lorrain et al., 2008; Martinez-Garcia et al., 2014; Yang and Li, 2017). Thus, reduced L_N_ in the High-B+FR is most likely due to increased proportion of far-red light in this treatment.

A significantly higher LA_s_ was observed in the High-B+FR compared to High-R and High-B treatments (Table 2). This means, leaf lamina of individual leaves expanded more in the High-B+FR treatment compared to other treatments. The plants in the High-B+FR likely exhibited proximity responses but were not “truly” exposed to shade during growth. Due to decreased L_N_ and reduced leaf layers (Figure 3), there likely was little intra-canopy shading and increased light interception in the High-B+FR. As plants did not experience shade or low light intensity after initiating shade responses, products of photosynthesis were available continuously to a small number of leaves, thereby resulting in expansion growth of individual leaves. This is the likely reason for increased LA_s_ in the High-B+FR than High-B. The LPR was not different between High-B and High-B+FR, but products of photosynthesis were distributed to relatively more number of leaves in the High-B, resulting in smaller LA_s_. Thus, the increased CA or LA_s_ in the High-B+FR than High-B is likely a consequence of reduced L_N_ and continued photosynthesis, albeit at a lower rate. Meng and Runkle (2019) and Zhen and Bugbee (2020) indicated that increased vegetative growth with far-red addition was related to increased canopy photon capture. Our results agree with the above two studies. In addition, our results point to the additional fact that increased SDW from increased CA in the High-B+FR is an indirect consequence of a negative effect of far-red light on L_N_ in lettuce.

### Plant Pigments

On a fresh weight basis, the levels of β-carotene and Chl were highest in the High-B, intermediate in the High-R, and lowest in the High-B+FR treatment (Table 3). Anthocyanins levels were not statistically different but trended similar to other pigments. The levels of β-carotene and Chl in the High-B was higher by 31 and 23%, respectively, than High-B+FR treatment. Pigment differences expressed on a leaf area basis were generally similar to those expressed on fresh weight basis. The level of β-carotene was highest in the High-B, intermediate in the High-R, and lowest in the High-B+FR (Table 3). The levels of anthocyanins and Chl were not statistically different when expressed on a leaf area basis. The levels trended lower in High B+FR. These results indicate that high percentage of blue light can increase pigments levels in romaine lettuce. Pigments were consistently lower in the High-B+FR than High-B in spite of exposure to relatively high percentage of blue light (42%) in the High-B+FR treatment. This indicates that far-red light has more influence on plant responses than blue light in the High-B+FR treatment. The Chl_(*a/b*)_ ratio was significantly lower in the High-B+FR compared to other treatments, while no differences were observed between the High-R and High-B treatments (Table 3). This suggests that lower level of Chl in the High-B+FR treatment is likely due to relatively larger decrease in the concentration of Chl_*a*_ than Chl_*b*_.

**FIGURE 5:**
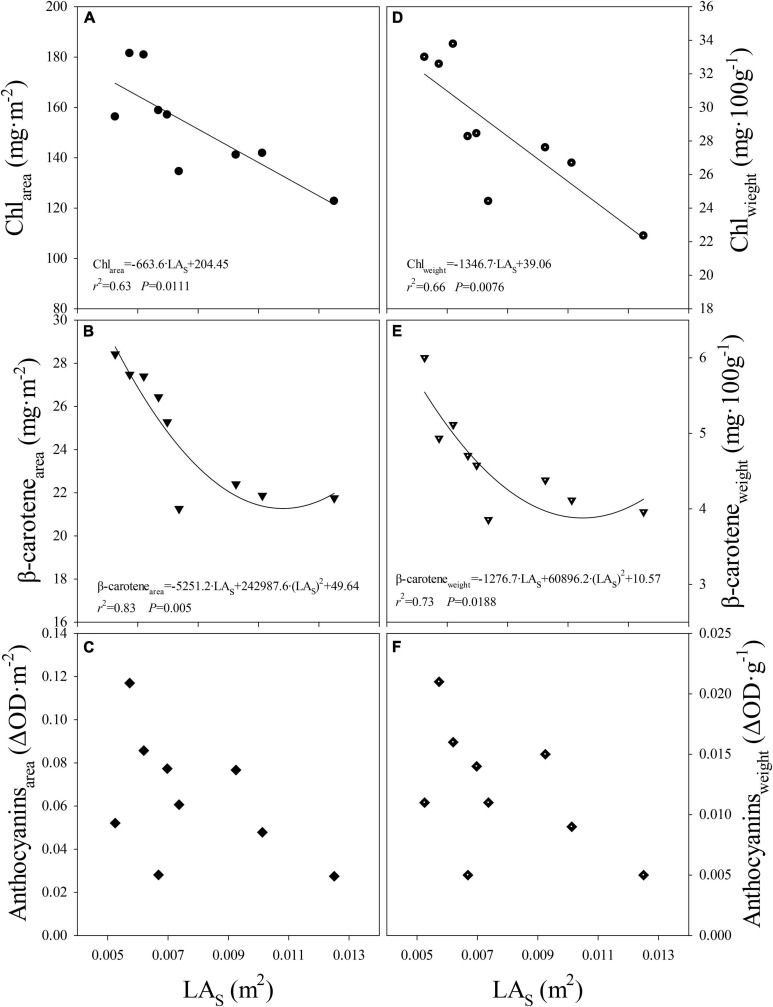
Relationship between **(A)** level of chlorophylls per unit leaf area (Chl_area_) and average area of a single leaf (LA_s_), **(B)** level of beta-carotene per unit leaf area (β-carotene_area_) and LA_s_, **(C)** level of anthocyanins per unit leaf area (Anthocyanins_area_) and LA_s_, **(D)** levels of chlorophylls per unit fresh weight (Chl_weight_) and LA_s_, **(E)** level of beta-carotene per unit fresh weight (β-carotene_weight_) and LA_s_, **(F)** level of anthocyanins per unit fresh weight (Anthocyanins_weight_) and LA_s_ in romaine lettuce. Data from three light treatments and three replications per treatment are shown. A linear regression was fitted to data in panels **(A,D)** and a quadratic regression was fitted to data in panels **(B,E)**.

When data from all light treatments were pooled, negative linear and quadratic relationships were observed between the level of Chl or β-carotene expressed on leaf area or fresh weight basis and LA_s_, respectively (Figures 5A,B,D,E). Although the response was not significant, similar trend was observed between anthocyanins levels per unit leaf area or fresh weight and LA_s_ (Figures 5C,F). This indicates that, regardless of light composition, pigment levels per unit leaf area decreased as LA_s_ increased. Further, this indicates that the differences in pigment levels can be explained mostly by changes in the LA_s_. In other words, levels of pigments were regulated likely at the individual leaf scale. As LA_s_ became smaller (e.g., High-B treatment), the pigment levels increased and as the LA_s_ became larger (e.g., High-B+FR treatment), a decrease in pigment levels was observed. This is likely because resources for leaf expansion and pigment synthesis share a common source, i.e., glucose from photosynthesis. For example, precursors of anthocyanin (e.g., malonyl CoA; Tanaka et al., 2008), and Chl, and β-carotene (e.g., pyruvate and glyceraldehyde 3-phosphate; Meier et al., 2011; Ruiz-Sola and Rodríguez-Concepción, 2012) are formed in glycolysis that uses glucose from photosynthesis as the substrate (Horton et al., 2002). However, more work is needed to further understand the involved biochemical mechanisms.

**FIGURE 6:**
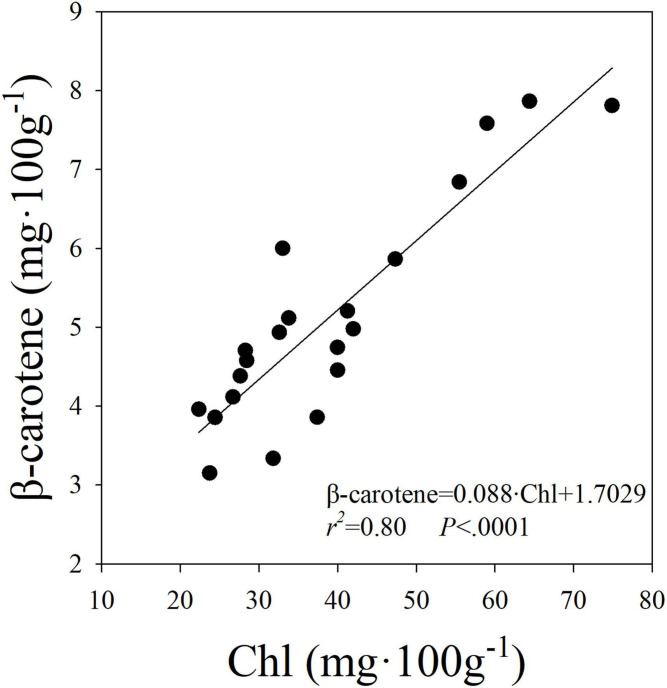
Linear relationship between the levels of beta-carotene (β-carotene) and chlorophylls (Chl) in romaine lettuce. A linear regression was fitted to data from three treatments and all seven replications from two experiments.

A linear and positive relationship was observed between the level of Chl and β-carotene expressed on fresh weight basis (Figure 6). These results suggest that the increase in the level of Chl was associated with an increase in the level of β-carotene in plants. Both Chl and β-carotene synthesis were shown to be upregulated by blue light (Fu et al., 2012; Tuan et al., 2017) and dependence of β-carotene synthesis on biosynthesis of Chl was previously reported (Bohne and Linden, 2002; Fu et al., 2012). Further, coordinated transcription of phytoene synthase and many isoprenoid biosynthesis genes was shown to be critical for regulating biosynthesis of carotenoids and Chl (Meier et al., 2011) and involvement of a STAY-GREEN protein for regulation of lycopene and β-carotene biosynthesis during ripening processes was reported in tomato (Luo et al., 2013). The linear relationship between β-carotene and Chl in our study suggests that the proportionate change in both pigments was not different among the light treatments in our study. Similar to our results, Härtel and Grimm (1998) indicated that β-carotene to Chl ratio remained mostly unchanged in tobacco under low and high light environments. These results indicate that factors that increase Chl (e.g., tissue nitrogen concentration) can likely increase β-carotene levels in leaves.

## Conclusion

**FIGURE 7:**
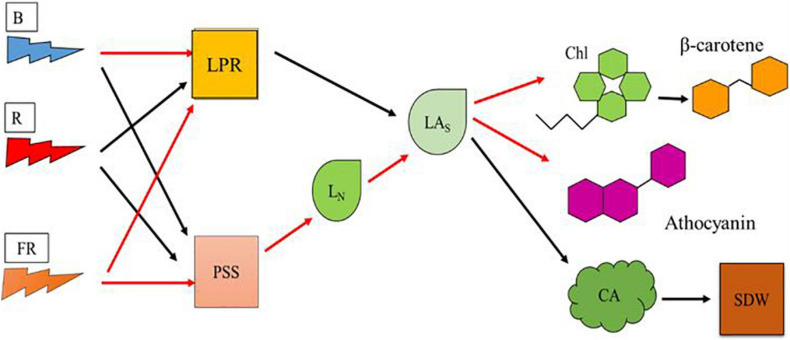
A model showing the effects of blue, red, and far-red radiation on physiological responses at cellular and leaf scales that affect vegetative growth and pigment synthesis in romaine lettuce. Model parameters include blue light (B), red light (R), far-red light (FR), leaf photosynthesis rate (LPR), phytochrome photostationary state (PSS), average leaf number per plant (L_N_), average area of individual leaf (LA_s_), chlorophylls level (Chl), beta-carotene level (β-carotene), anthocyanins level, canopy area (CA), and shoot dry weight (SDW). Black and red arrows indicate positive and negative relationships, respectively, between two parameters.

Our objectives were to understand the effects of high-energy blue and low-energy far-red on vegetative growth and pigment synthesis in plants. In our study, effects of light composition on vegetative growth and pigment synthesis appear to be mediated by L_N_ and LA_s_ (Figure 7). Romaine lettuce plants provided with high proportion of blue light showed decreased growth and increased levels of Chl and β-carotene. Plants provided with high proportion of blue radiation showed decreased LPR, likely due to lower photosynthetic efficiency of blue light. Addition of far-red light to high proportion of blue light resulted in relatively more photomorphogenic effects on plants. We observed morphological changes including lower L_N_ and increase in the LA_s_ in plants exposed to far-red light. Addition of low-energy far-red light to high-energy blue light did not enhance LPR. When L_N_ increases without a change in LPR as in the High-B compared with High-B+FR, the products of photosynthesis are distributed to larger number of leaves thereby resulting in smaller LA_s_. Whereas when L_N_ decreases without change in LPR as in High-B+FR compared to High-B, the products of photosynthesis will be allocated to a fewer number of leaves thereby resulting in larger LA_s_. The High-R treatment showed positive effects on both LPR and L_N_, resulting in LA_s_ intermediate to that of the High-B and High-B+FR treatments. Larger LA_s_ in the High-B+FR likely resulted in larger CA (and likely increased light interception) and SDW. Whereas, intermediate LA_s_ coupled with increased LPR in High-R, likely resulted in increased SDW. In contrast, smaller LA_s_ and decreased LPR likely resulted in smaller CA and SDW in the High-B. Pigment levels increased with decreasing LA_s_ and decreased with increasing LA_s_. Because of this, the levels of Chl and β-carotene (and anthocyanins likely) were highest in the High-B, followed by the High-R, and lowest in the High-B+FR treatment. The results from this study indicate that high-energy blue and low-energy far-red light affect the number and expansion of individual leaves differently, thereby influencing both vegetative growth and pigment synthesis in lettuce. We hope that the information generated in this study can aid in increasing our understanding of plant responses to high-energy blue and low-energy far-red radiation and optimizing lighting environment in vertical farms.

## Data Availability Statement

The raw data supporting the conclusions of this article will be made available by the authors, without undue reservation.

## Author Contributions

YK performed the experimental design, experimentation, data collection, data analyses, and manuscript preparation. KN contributed to project leadership, funding, experimental design, data analyses, and manuscript preparation. Both authors contributed to the article and approved the submitted version.

## Conflict of Interest

The authors declare that the research was conducted in the absence of any commercial or financial relationships that could be construed as a potential conflict of interest.
